# Optimizing germination conditions of Ghaf seed using ZnO nanoparticle priming through Taguchi method analysis

**DOI:** 10.1038/s41598-024-67025-6

**Published:** 2024-07-10

**Authors:** Dali V. Francis, Abdul Subhan, Abdel-Hamid I. Mourad, Abdelmoneim K. Abdalla, Zienab F. R. Ahmed

**Affiliations:** 1 Integrative Agriculture Department, College of Agriculture and Veterinary Medicine, United Arab Emirates University, 15551 Al Ain, UAE; 2 Mechanical and Aerospace Engineering Department, College of Engineering, United Arab Emirates University, 15551 Al Ain, UAE; 3 Food Science and Technology Department, College of Agriculture, South Valley University, Qena, 83523 Egypt

**Keywords:** Ghaf, Nanoparticle, Seed priming, Optimization, Validation tests, Taguchi, Computational biology and bioinformatics, Physiology, Plant sciences

## Abstract

Ghaf, a resilient tree in arid environments, plays a critical role in ecological restoration, desertification mitigation, and cultural heritage preservation. However, the seeds’ inherent challenges, notably their hard outer coating restricting germination, emphasize the pressing need for innovative strategies. This work aimed to investigate the optimization of Ghaf seed germination process through seed priming with ZnO nanoparticles treatment (duration (t), concentration (c), temperature (T), and agitation (a), employing the Taguchi method for efficient experimental design. Furthermore, the study includes Analysis of Variance (ANOVA), analysis for the regression model to assess the significance of predictor variables and their interactions, thereby strengthening the statistical validity of our optimization approach. Notably, it revealed that concentration is a pivotal influencer in optimization of Ghaf seed germination. The results showed that the concentration of ZnO nanoparticles has no linear relation with any other parameters. To verify the outcomes, validation tests were performed utilizing the predicted optimal parameters. The observed low error ratio, falling within the range of 1 to 6%, confirmed the success of the Taguchi methodology in identifying optimal levels of the factors chosen. Significantly, ZnO-primed seeds showcased a remarkable enhancement in Ghaf seed germination, increasing from 15 to 88%. This study introduces a novel approach utilizing ZnO nanoparticle treatment optimized through the Taguchi method, significantly enhancing seed germination rates of Ghaf seeds and offering a promising avenue for sustainable agricultural practices in arid environments.

## Introduction

Ghaf seeds pose inherent challenges in germination, presenting a significant obstacle to the propagation of this resilient desert tree^1^. The seeds’ hard outer coating, characteristic of arid environment adaptations, hinders their prompt germination, with only approximately 15% naturally responsive after water immersion^2^. This low germination percentage restricts the potential expansion of Ghaf tree populations, impacting ecological restoration efforts and diminishing the tree's vital role in arid ecosystems. Consequently, there is a compelling need to devise innovative strategies to overcome the dormancy barriers of Ghaf seeds and enhance their germination rates^1^.

The significance of improving Ghaf (*Prosopis cineraria*) seed germination in the UAE is emphasized by its contribution to ecological restoration, desertification mitigation, preservation of cultural heritage (as the national tree of UAE), and support for sustainable resource utilization, including traditional medicine^3,4^.

Developing innovative methods to enhance seed germination is essential for promoting sustainable crop cultivation^5,6^. Various methods for seed priming have been investigated to boost the synchronicity and speed germination such as chemical-, hydro-/osmo-, bio-, physical- and nutrient-priming especially nano-element^7–9^. With seed priming recognized as a pre-sowing treatment offering promise in optimizing germination conditions, CuO and ZnO nanoparticles, recognized for multifaceted roles in plant growth, emerge as a compelling agent for this purpose^10^. The seeds that undergo imbibition post-treatment are referred to as 'responded seeds, which subsequently undergo germination. During the priming of Ghaf seeds, the responded seeds exhibit changes in color, length, width, and weight^2^. There are several factors affecting seed germination and break the dormancy of different crops’ seeds in response to priming treatments, for instance temperature, light, duration, and soil moisture^3^. Investigation of such multiple factors along with a seed priming treatment at the same time is still a challenge.

Employing a methodology for efficient experimental design such as Taguchi method may facilitates the simultaneous exploration of multiple factors, providing a systematic approach to discern optimal conditions for seed germination improvement^11^. The Taguchi method's unique strength lies in its efficiency in navigating complex interactions among chosen factors^12^, thereby offering a comprehensive understanding of their individual and collective impacts on seed germination of crops such as Ghaf.

Moreover, this research contributes significantly to broader applications in agriculture, environmental conservation, and cultural preservation. By demonstrating the effectiveness of ZnO nanoparticle priming in enhancing seed germination rates, particularly in challenging arid environments, this study not only addresses critical regional challenges but also aligns with global sustainability initiatives. The findings underscore the potential of innovative nanotechnological approaches to promote resilient crop cultivation, mitigate desertification, and preserve biodiversity. This dual impact makes it a pivotal contribution to both local agricultural practices and broader international efforts towards sustainable development and environmental stewardship^3^.

Therefore, the present study objective was to enhance the percentage of responded Ghaf seeds to priming treatment by ZnO nanoparticles. Beyond that, the research introduced valuable insights into nanotechnology-driven seed enhancement, aiming to reveal novel strategies using the Taguchi method to optimize seed priming methodologies. This collective effort propels towards a more sustainable agricultural future by leveraging the distinct potential of ZnO nanoparticles to enhance the germination potential of Ghaf seeds.

## Results

### Characterization of ZnO nanoparticle

**Figure 1:**
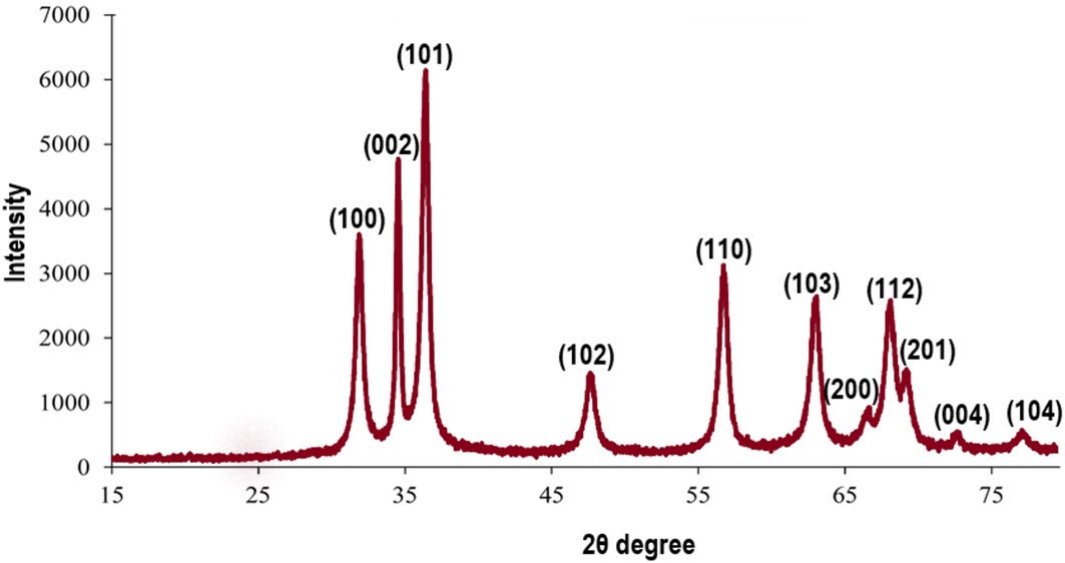
X-ray diffractogram of chemically synthesized ZnO Nanoparticles.

**Figure 2:**
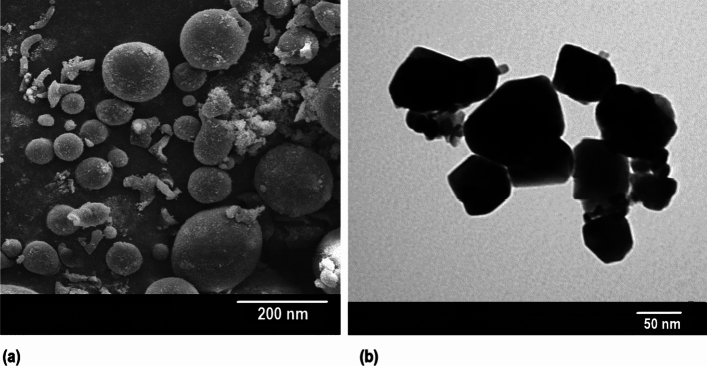
(**a**) SEM analysis of chemically synthesized ZnO nanoparticle using JEOL JSM-7600F FEG-SEM at 20 kV with scale bar of 200 nm (**b**) TEM analysis of chemically synthesized ZnO nanoparticle by JOEL 2100 at 10kv with a scale bar of 50 nm.

**Figure 3:**
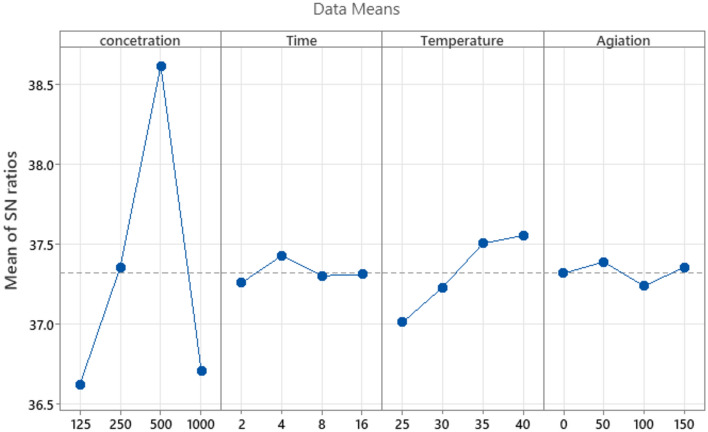
Main effect plot for S/N ratio of concentration, time, temperature and agitation for on percentage of responded Ghaf seed.

The XRD graph shown in Figs. 1, 2, 3, and Table1 present a detailed characterization of ZnO nanoparticles. Distinctive 2θ values (31.94, 34.64, 36.42, 47.83, 56.85, 62.93, and 68.2) in Fig. 1, corresponding to zinc oxide diffraction (100, 002, 101, 102, 110, 103, 112), authenticate the crystalline structure^13^. Employing the Debye–Scherrer equation, the average grain size^14^ was determined at 42.64 nm (± 3) (Table 1), further verification by scanning electron microscopy (SEM), revealed the spherical morphology of ZnO nanoparticle, providing further confirmation of their size being less than 100 nm (Fig. 2a). Additionally, transmission electron microscopic (TEM) analysis in Fig. 2b confirmed a particle size range of 50–100 nm.

**Table 1:** Crystallite size of synthesized ZnO nanoparticles as determined from XRD analysis.

Peak position (2θ)	FWHM (θ)	Crystallite size (nm)	Average crystallite size (nm)
31.9497	0.2157	37.9548	42.13 ± 5
34.64986	0.241	34.2278	
36.42525	0.2614	30.3541	
47.83306	0.1431	60.0663	
56.85048	0.1738	48.0504	

For this experiment, a sample of Ghaf seeds weighing ten grams, equivalent to approximately (350 ± 6) seeds in triplicates, was used. Following the imbibition process, significant alterations were observed in the shape, weight, and color of the Ghaf seeds. The primary response parameter studied is the percentage of responded seeds. The numbers of responded seeds are counted in each run to calculate the percentage of responded seeds.

### Identification of optimum factors and their levels

The experimental design, structured in accordance with the Taguchi orthogonal array (L16), was executed to investigate the impact of varied physico-chemical parameters on seed germination. Table 2 encapsulates the response values for mean responded seed and Signal to Noise (S/N) ratio, with all experimental trials meticulously conducted in triplicates to ensure result accuracy and reliability. Observations across the experimental runs revealed notable fluctuations in responded seed percentages. Specifically, experimental run 16 (C4T4t1A2) exhibited the minimum responded seed percentage, while conversely, experimental run 10 (C3T2t4A2) demonstrated the highest percentage at 88% (Table 3). These findings underscore the discernible influence of combined physico-chemical parameters during incubation on the percentage of responded seeds and subsequent seed germination. The analysis of these outcomes provides valuable insights into the nuanced effects of experimental conditions on critical response variables. Concentration consistently stood out as the most favorable at Level 3, showcasing the highest S/N ratio in the main effect plot (Fig. 3). Similarly, temperature displayed optimal performance at Level 3, emphasizing its significance, while time peaked at Level 2, and Agitation at Level 1.

**Table 2:** The selected experimental trials designed using the Taguchi method for enhancing the germination of Ghaf seeds using ZnO nanoparticle.

Experimental run	Concertation (ng/mL)	Time (Hr)	Temperature (ͦC)	Agitation (RPM)
1	125	2	25	0
2	125	4	30	50
3	125	8	35	100
4	125	16	40	150
5	250	2	30	100
6	250	4	25	150
7	250	8	40	0
8	250	16	35	50
9	500	2	35	150
10	500	4	40	100
11	500	8	25	50
12	500	16	30	0
13	1000	2	40	50
14	1000	4	35	0
15	1000	8	30	150
16	1000	16	25	100

**Table 3:** Experimental design using Taguchi L16 orthogonal array design (OA) and the percentage of responded Ghaf seeds and Signal–noise ratio for each factor level combination designed.

Experimental run	Concertation (ng/mL)	Time (Hr)	Temperature (^º^C)	Agitation (RPM)	S/N ratio	Responded seeds (%)	Std dev
1	125	2	25	0	36.25	65	4
2	125	4	30	50	36.52	67	7
3	125	8	35	100	36.77	69	9
4	125	16	40	150	36.90	70	3
5	250	2	30	100	37.15	72	5
6	250	4	25	150	37.276	73	4
7	250	8	40	0	37.38	74	7
8	250	16	35	50	37.61	76	6
9	500	2	35	150	38.59	85	3
10	500	4	40	100	38.89	88	6
11	500	8	25	50	38.38	83	5
12	500	16	30	0	38.59	85	5
13	1000	2	40	50	37.03	71	7
14	1000	4	35	0	37.03	71	4
15	1000	8	30	150	36.66	68	6
16	1000	16	25	100	36.12	64	5

The main effect plot and response table for S/N ratio provide a focused assessment of the optimization study outcomes^15^, specifically examining concentration, time, temperature, and agitation. In the S/N ratio table, where larger values are preferable, each factor is evaluated at four levels (1 to 4), presenting the corresponding S/N ratio. The Delta values represent the difference between the maximum and minimum S/N ratio for each factor, indicating the degree of improvement across levels^16^. Concentration emerges as the top performer with a Delta of 2.00 Table 4, showcasing the most significant improvement among factors. Temperature follows with notable improvement, while time and agitation show marginal changes.

**Table 4:** Mean signal–noise ratio by factor level for concentration, time, temperature and agitation on percentage of responded Ghaf seeds.

Mean signal–noise ratio				
Level	Concentration	Time	Temperature	Agitation
1	36.61	37.25	37.01	37.31
2	37.35	37.43	37.23	37.39
3	38.61	37.30	37.50	37.23
4	36.71	37.31	37.55	37.35
Delta	2.00	0.17	0.54	0.15
Rank	1	3	2	4

These response tables emphasize concentration's pronounced influence on the optimization study, seen in both S/N ratio and mean response values. The findings offer valuable guidance for prioritizing and refining optimization parameters, with concentration identified as a key driver of improvement.

### ANOVA and regression analysis

**Figure 4:**
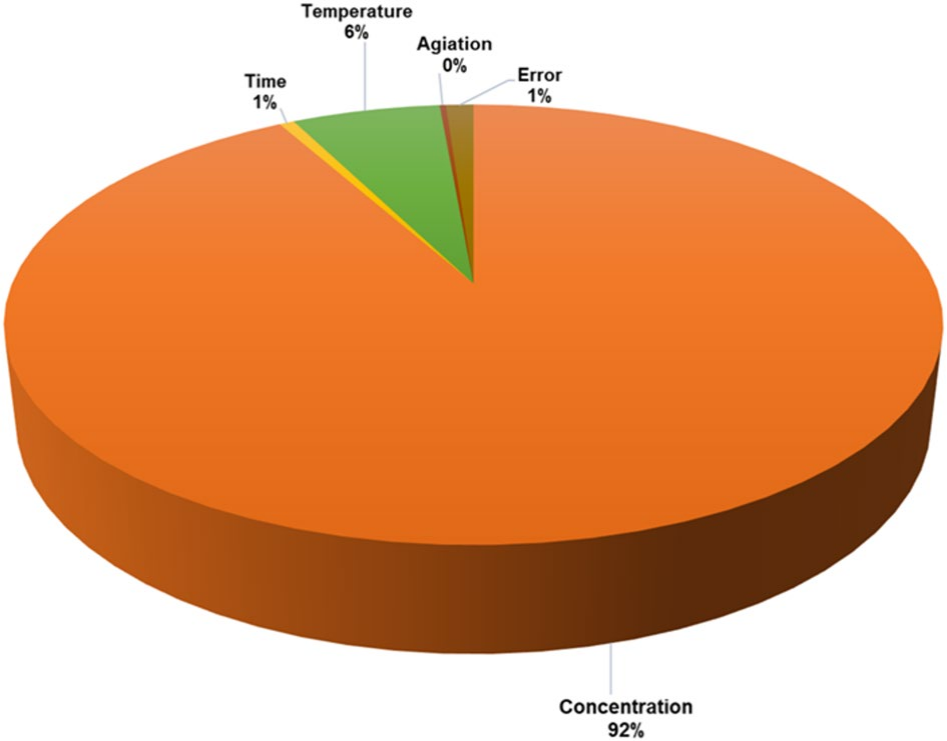
ANOVA analysis for percentage of contribution of concentration, time, temperature, and agitation on percentage of responded Ghaf seeds.

Analysis of Variance (ANOVA) was employed to evaluate the effects of incubation parameters on the improvement of Ghaf seed germination rates. Results indicated that the concentration of ZnO nanoparticles played a pivotal role, contributing 92% to the observed variability in germination rates. Temperature exhibited a moderate impact at 6%, whereas both time and agitation showed minimal effects of 1% each. The residual variance, attributed to errors in measurement, accounted for the remaining 1%. These findings underscore the critical influence of ZnO nanoparticle concentration in optimizing the germination process of Ghaf seeds (Fig. 4)*.*

Table 5 presents the results of the ANOVA and regression analysis for the optimization study, offering a breakdown of the contributions of different factors to the observed variability in the response variable. The Regression analysis, comprising concentration, time, temperature, and agitation, is statistically significant (p ≤ 0.014) and accounts for 98.86% of the total variability. Concentration stands out as the primary influencer, demonstrating substantial significance (p ≤ 0.002) and contributing 91.88% to the model's explanatory power. This underscores concentration's pivotal role in the optimization process. On the other hand, time, temperature, and agitation exhibit comparatively lower impacts. Temperature contributes significantly to the model, explaining 6.12% of the variability (p ≤ 0.100), while time and agitation show minimal influence. Table 5 presented F-test results that validates the statistical significance of the regression model, highlighting concentration as the major driver and emphasizing its crucial role in explaining the observed variability in the response variable within the optimization study.

**Table 5:** ANOVA output on contribution of concentration, time, temperature, and agitation on percentage of responded Ghaf seeds (at 95% confidence level).

Source	DF	Seq SS	Contribution	Adj SS	Adj MS	F-value	P-value
Regression	12	842.75	98.86%	842.75	70.22	21.75	0.01
Concentration	3	783.18	91.88%	783.18	261.063	80.85	0.00
Time	3	5.18	0.61%	5.18	1.729	0.54	0.690
Temperature	3	52.18	6.12%	52.18	17.396	5.39	0.100
Agitation	3	2.187	0.26%	2.18	0.729	0.23	0.873
Error	3	9.687	1.14%	9.68	3.229		
Total	15	852.438	100.00%				

### Analysis of interelation between the factors

**Figure 5:**
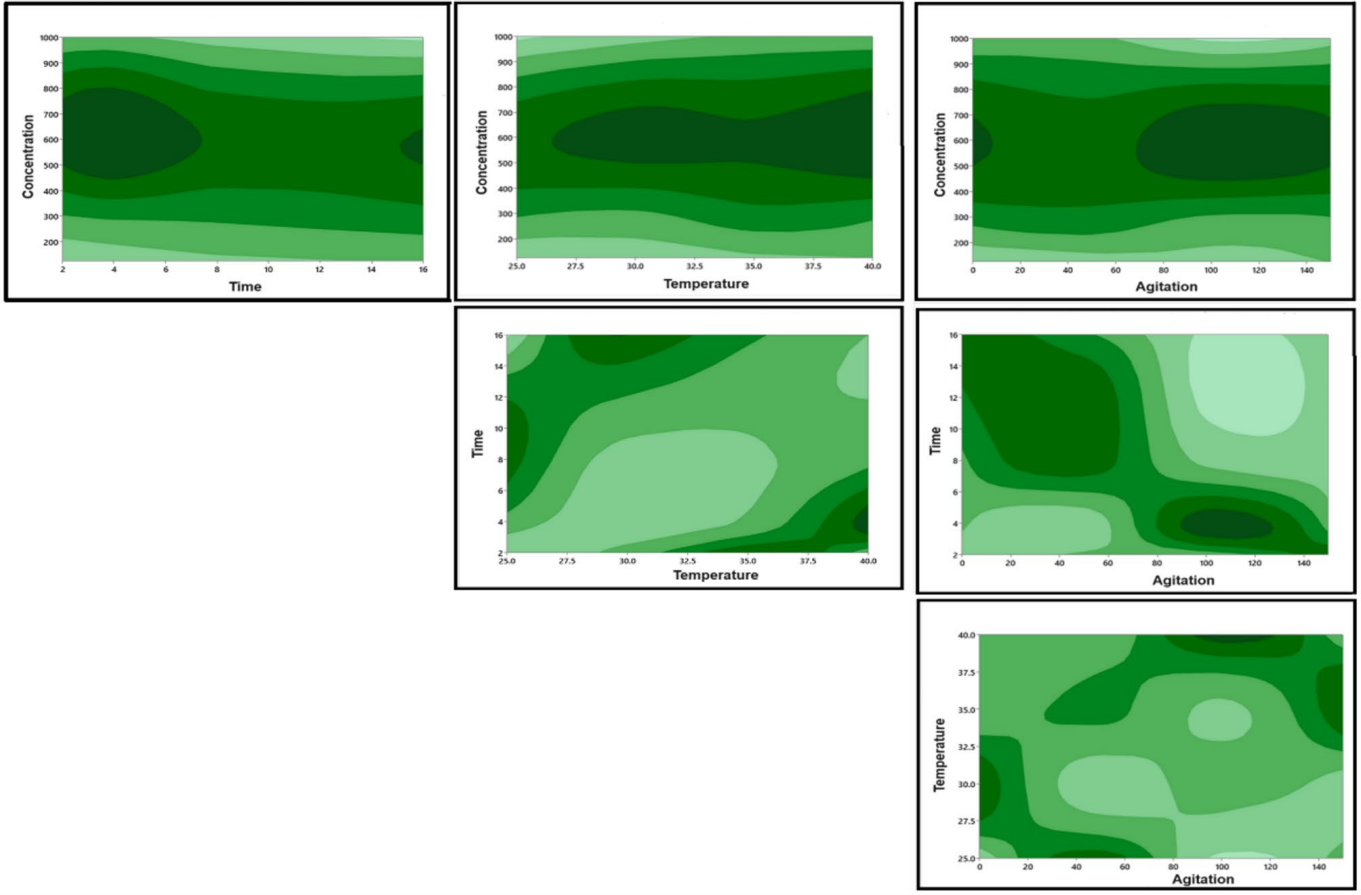
Two-dimensional contour plots illustrate the influence of concentration, time, temperature and agitation on percentage of responded Ghaf seeds.

The analysis of the contour plot (Fig. 5) reveals that concentration exhibits a lack of interaction with other parameters, such as temperature, time, and agitation. The contour lines corresponding to concentration remain consistently parallel, indicating that changes in concentration do not significantly impact the response variable differently at various levels of temperature, time, or agitation. On the contrary, a robust interaction is evident between the factors of time, temperature, and agitation. The contour lines associated with the combinations of these variables intersect and exhibit non-parallel patterns. This indicates that alterations in one of these factors have a varying impact on the response depending on the levels of the other two factors^17^. The observed strong interaction among time, temperature, and agitation underscores the complexity of their combined effects and emphasizes the need for a comprehensive understanding of their interplay in optimizing the system. Optimizing seed germination outcomes, thereby enhancing the precision and efficacy of our experimental approach.

### Predicting the best factor for increased % of responded Ghaf seeds

The regression model, with a low standard error (S = 1.79699) and a high R-squared value (98.86%), effectively explains nearly 99% of the variability in responded seeds based on concentration, time, temperature, and agitation.

The regression analysis reveals a predictive model for the number of responded seeds based on the factors of concentration, time, temperature, and agitation. The regression Eq. (1):

1 $$ {\text{Responded seeds}} = {64}.{56 } + 0.0 {\text{concetration}}\_{125 } + {6}.00 {\text{concetration}}\_{25}0 \, + {17}.{5}0 {\text{concetration}}\_{5}00 \, + 0.{75} {\text{concetration}}\_{1}000 \, + 0.0 {\text{Time}}\_{2 } + {1}.{5}0 {\text{Time}}\_{4 } + 0.{25} {\text{Time}}\_{8 } + 0.{5}0 {\text{Time}}\_{16 } + 0.0 {\text{Temperature}}\_{25 } + {1}.{75} {\text{Temperature}}\_{3}0 \, + {4}.00 {\text{Temperature}}\_{35 } + {4}.{5}0 {\text{Temperature}}\_{4}0 \, + 0.0 {\text{Agiation}}\_0 \, + 0.{5}0 {\text{Agiation}}\_{5}0 \, - 0.{5}0 {\text{Agiation}}\_{1}00 \, + 0.{25} {\text{Agiation}}\_{15}0, $$

The regression equation represents the relationship between the responded seeds and the specified levels of concentration (125, 250, 500, and 1000) ng/mL, time (2, 4, 8, and 16) hrs., temperature (25, 30, 35, and 40) ͦ C, and agitation (0, 50, 100, and 150) RPM. Each coefficient indicates the contribution of the corresponding factor level to the number of responded seeds.

**Figure 6:**
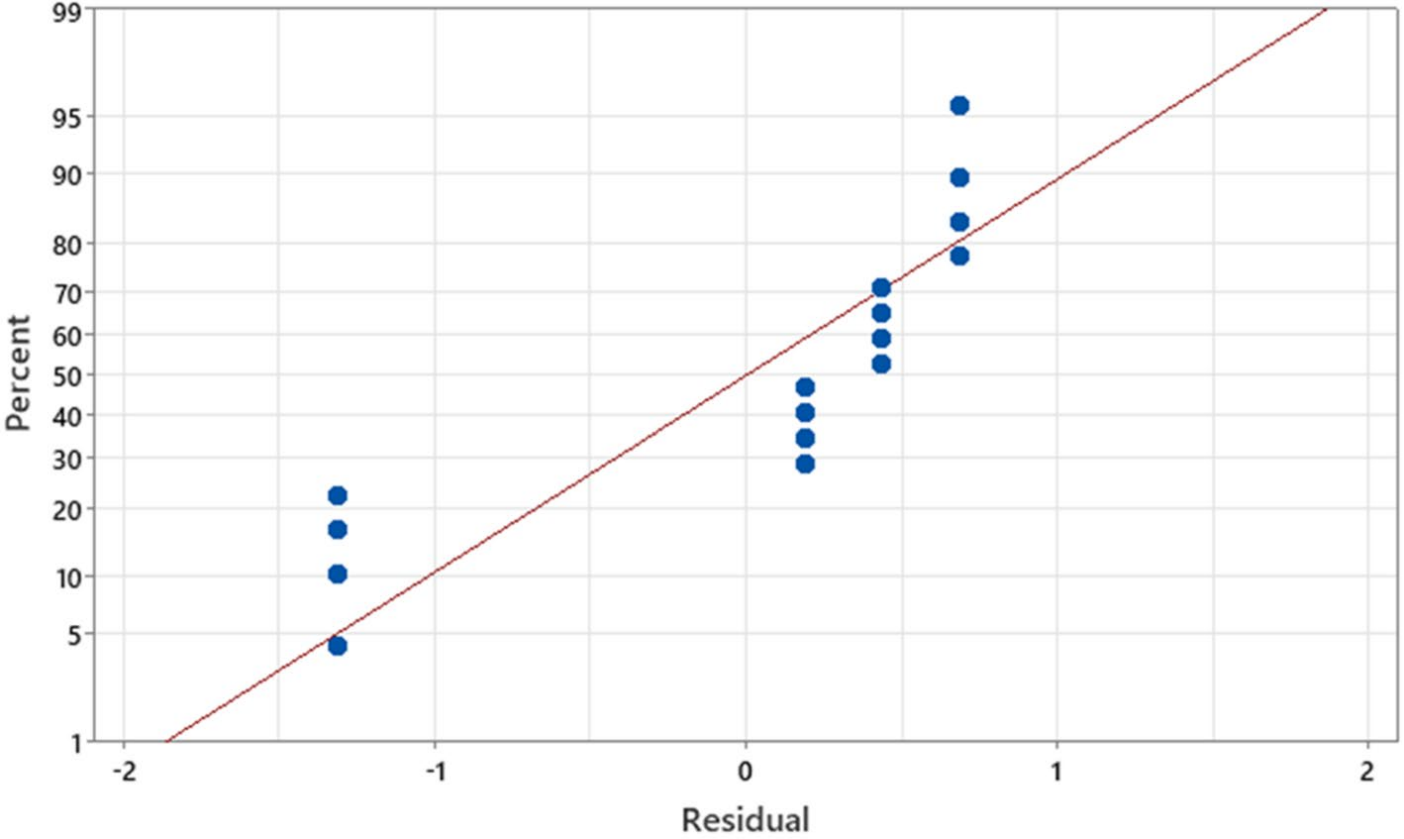
Normal probability plot of residuals for the regression analysis of the percentage of responded seeds to concentration, time, temperature, and agitation.

The Normal Probability Plot (Fig. 6) assesses the normality of the residuals from the regression analysis by comparing the residuals to their expected values under a normal distribution. In this analysis, the residuals closely follow the red line, suggesting that the assumption of normality is reasonably met. This validation of normality enhances the credibility of our regression model, which has a high R-squared value (98.86%), indicating that the model’s predictions and the statistical inferences drawn are reliable. The normal distribution of residuals is crucial for ensuring the accuracy and robustness of the Taguchi regression analysis applied in optimizing the germination conditions for Ghaf seeds.

Regression equations were used to compute the anticipated percentages of responded seeds for each trial run and predict the impact of alterations in parameters on the dependent variables. The corresponding predicted values for the percentage of responded seeds in certain experimental runs are detailed in Table 4. An alternative approach for forecasting the dependent variables involves the formulation of equations through the Taguchi method. Consequently, the Taguchi-predicted values for multiple S/N ratios at the optimal factor level (ε0) were also determined using Eq. (2).

2 $$ {\upvarepsilon }0\, = {\upvarepsilon }m\, + \sum i = 1j\,\,\left( {{\upvarepsilon }i - {\upvarepsilon }m} \right). $$

Here, ε0 represents the prediction, εm denotes the total mean S/N ratio, εi signifies the mean S/N ratio at the optimal level, and j corresponds to the number of input process parameters^18^.

### Validation of the predicted equations and confirmatory analysis

**Figure 7:**
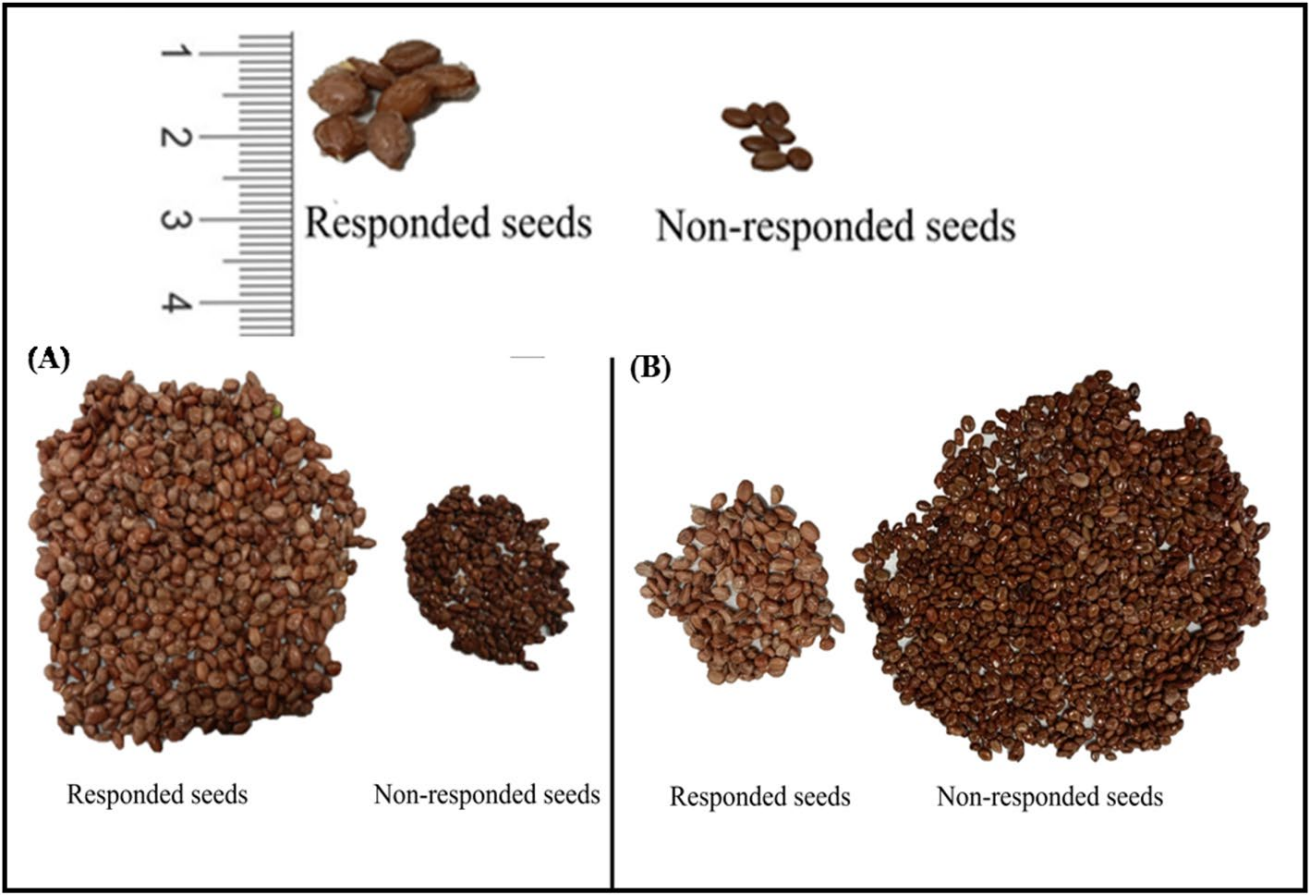
Ghaf seeds treated with optimal parameter combination of 500 ng/ml concentration, 40 °C temperature, 4 h duration, and 50 RPM agitation (C3t2T4a2) (**A**), and seeds treated with water (control) (**B**).

Verification of the proposed experimental design's validity is crucial to substantiate the expected enhancement in the process response through the optimal parameters identified by the matrix test. Five runs (1, 4, 8, 12 & 16) were randomly selected from the L16 Taguchi Orthogonal Array (OA), in addition to a run utilizing the optimized levels of incubation parameters (C3t2T4a2), for the purpose of validating both the regression and Taguchi models. The predicted values from both Taguchi and regression analyses are same, demonstrating consistency in their predictions. Each experimental run was conducted in triplicate, and the average percentage of responded seeds was compared with the predicted values. Table 6 presents the comparative data between actual experimental values and predicted values for particle size and PDI, as determined by the Taguchi method and regression prediction equation. The minimal error observed (less than 6% of responded seeds) in the comparison between experimental and predicted values underscores the efficacy of mathematical modeling in forecasting optimal incubation parameters for enhanced seed germination. A confirmatory analysis given in Fig. 7A illustrates the emergence of responded seeds following treatment with the optimized parameter combination of 500 ng/ml concentration, 40 °C temperature, 4 h duration, and 50 RPM agitation (C3t2T4a2). In contrast, Fig. 7B portrays the seeds treated with a water control.

**Table 6:** Predicted values and confirmation test results by Taguchi method for random runs and optimized combination levels for the percentage of responded Ghaf seeds.

RUN	Concentration (ng/ml)	Time (hours)	Temperature (^o^celsius)	Agitation (RPM)	Actual	Predicted	Error %
1	125	2	25	0	65	63	3.0769
4	125	16	40	150	70	71	− 1.429
8	250	16	35	50	76	75	1.3158
12	500	16	30	0	85	83	2.3529
16	1000	16	25	100	64	65	− 1.563
C3t2T4a2	500	4	40	50	84	89	− 5.952

C3t2T4a2: combination of 500 ng/ml concentration, 40 °C temperature, 4 h duration, and 50 RPM agitation.

## Discussion

The chemically synthesized ZnO nanoparticles exhibited a crystalline structure, confirmed by XRD analysis. The Debye–Scherrer equation determined an average grain size of 42.64 nm, while scanning TEM and SEM analyses revealed a spherical morphology and a particle size range of 50–100 nm. The effectiveness of ZnO nanoparticle in enhancing seed germination is evident, yet the concentration plays a pivotal role. While these nanoparticles have been demonstrated to promote germination, excessive concentrations pose potential harm.

The experimental design is founded on the Taguchi approach, utilizing the orthogonal array to efficiently explore the interactions between factors and levels while minimizing the number of experiments^19^. The Taguchi method's orthogonal arrays enable the exploration of various parameter combinations in a relatively concise set of experimental runs. This efficiency is paramount when dealing with complex interactions among factors^20^. The method's ability to identify the most influential factors and their optimal levels is pivotal for targeted and resource-effective optimization^21^. Furthermore, Taguchi analysis provides statistical rigor, allowing for the quantification of parameter effects and the determination of statistically significant factors, enhancing the credibility of the findings. Additionally, the robustness testing inherent in the Taguchi method ensures that the identified optimal conditions remain effective in the face of minor variations or uncertainties. Ultimately, the Taguchi method serves as a powerful tool not only for understanding the factors influencing Ghaf seed germination but also for guiding the development of practical and optimized seed priming protocols with ZnO nanoparticle for real-world applications. Utilizing a full factorial design would entail conducting 256 runs in triplicate (i.e. 768 runs) to pinpoint the optimal combination of selected incubation parameters. In contrast, the Taguchi orthogonal array (OA) design offers a more streamlined approach to configuring experimental parameters compared to the factorial method, significantly reducing the number of experimental runs to 16. This method not only minimizes the experimental cost and time but also maintains efficiency in exploring key factors for optimization^22^.

The S/N Ratios play a pivotal role in assessing key factors in this study, revealing essential insights into performance^23^. A higher S/N ratio signifies superior performance, and our analysis identified distinct trends^24^, aligning with our goal of enhancing the germination percentage of Ghaf seeds. This formula encapsulates the essence of our approach, providing a quantitative measure of the success of our optimization efforts.

The Delta values quantified the variation within each factor, elucidating their impact on system performance^25^. The delta (Δ) value was determined by subtracting the lowest S/N from the highest S/N for each factor. Higher delta value signifies higher variation. Hence based on the delta value the concentration of ZnO nanoparticle is the most influential parameter followed by temperature, time and agitation. The Analysis of Variance (ANOVA) , a robust statistical approach allowed for the systematic exploration of key incubation parameters, enabling a comprehensive understanding of their individual and collective impacts on the germination process^26^.ANOVA results emphasize the statistical significance of the regression model. Concentration stands out as the major influencer, explaining 91.88% of the model's explanatory power (p ≤ 0.002), underscoring its pivotal role in the optimization process. Conversely, time, temperature, and agitation exhibit lower impacts, with temperature contributing 6.12% (p ≤ 0.100). The high F-value and low p-value for concentration suggest a strong and statistically significant relationship between concentration levels and the variability in the response variable. Studies suggests that nanoparticle may enhance seed germination mechanisms by potentially increasing water absorption^27^, improving seeds' capacity to absorb and utilize water and nutrients^28^, elevating nitrate reductase enzyme levels^29^, bolstering seed antioxidant systems to mitigate oxidative stress by reducing superoxide radicals and malonyldialdehyde content^30^, and boosting the activities of certain enzymes such as superoxide dismutase, ascorbate peroxidase, guaiacol peroxidase, and catalase^31^. Nanoparticle concentration significantly impacts seed germination by altering biochemical processes, making it the most influential factor in the study^32^. The concentration of ZnO nanoparticles emerges as the pivotal influencer in optimizing Ghaf seed germination due to its direct and profound impact on biochemical processes critical to seed viability and growth. Unlike factors such as temperature, time, and agitation, which influence external conditions during germination, nanoparticle concentration directly affects the quantity of nanoparticles available for interaction with seed tissues. This interaction can modulate physiological processes such as water uptake, nutrient absorption, and enzymatic activities within the seed^10,33^. Higher nanoparticle concentrations typically provide more active sites for these interactions, thereby potentially enhancing seed germination rates^34^. Consequently, the concentration of ZnO nanoparticles assumes centrality in this study as it fundamentally alters internal seed processes necessary for successful germination, distinguishing it as the most influential parameter among the factors investigated.

The intricate relationships between concentration, time, temperature, and agitation are further elucidated through the developed regression equation. The linear regression analysis aimed to model these relationships, capturing any linear or polynomial dependencies among the variables. The predicted values obtained through Taguchi OA experiments, executed with Minitab 19.0 software, were systematically compared with the outcomes derived from the linear regression equations^35^. This comparison was further validated through experimental observations, and the efficacy of the developed regression models was evaluated through statistical measures.

The potential of the regression models to explain the variability observed in Ghaf seed germination rates was quantitatively assessed using the coefficient of determination (R^2^)^36^. This methodological approach not only identified the key parameters influencing the enhancement of Ghaf seed germination but also validated the predictive capabilities of the developed regression models through rigorous statistical and experimental analyses.

The coefficients in the equation not only quantify the individual contributions of each factor level but also capture the collective impact of these factors on the responded seed count. The positive and negative coefficients signify the directionality and magnitude of these effects, offering a nuanced understanding of the optimization process^37^. The constant term (64.56) serves as a baseline, providing context for seed response when all factors are at their reference levels^38^.

The contour plot analysis adds depth to the discussion, revealing distinct patterns of interaction between factors. Notably, concentration demonstrates a lack of interaction with other parameters, maintaining parallel contour lines. This suggests that changes in concentration do not significantly alter the response variable in conjunction with variations in temperature, time, or agitation. In contrast, a robust interaction is observed among time, temperature, and agitation, as indicated by non-parallel contour lines. This emphasizes the need for a comprehensive understanding of the combined effects of these factors for effective optimization.

The predictive capabilities of the regression model are highlighted in the discussion, emphasizing its utility in anticipating the percentage of responded seeds for each experimental run. The comparison of predicted values with actual experimental outcomes, as depicted in Table 4, underscores the model's accuracy and reliability. The minimal error observed (< 6% of responded seeds) signifies the effectiveness of mathematical modeling in forecasting optimal incubation parameters, further validating the predictive power of the developed regression equation.

Moreover, the application of the Taguchi method offers a systematic and efficient approach to optimizing germination conditions. The method's ability to explore multiple factors simultaneously, using orthogonal arrays, facilitates a comprehensive understanding of the individual and collective impacts of the chosen parameters. The "larger the better" S/N ratio criterion ensures the quality of experimental outcomes, and the Delta values provide insights into the degree of improvement across factor levels. These aspects collectively contribute to the robustness and reliability of the optimization study.

This study focuses on optimizing parameters to enhance Ghaf seed germination using the Taguchi method, with the goal of establishing a green canopy in desert regions. Ghaf trees are uniquely adapted to harsh climates, making them suitable for combating desertification and increasing oxygen production. However, seed dormancy poses a significant challenge to achieving consistent germination rates and uniform plant growth. Addressing these issues is crucial as inconsistent germination patterns can lead to uneven plant growth. Given Ghaf trees' potency in harsh conditions, once established, they require minimal maintenance. This resilience underscores their potential as a sustainable solution for enhancing green cover in desert areas. By tackling seed dormancy and improving germination consistency, this initiative aims to promote a robust Ghaf tree population, contributing to environmental stability and biodiversity conservation in arid regions.

## Conclusion

In conclusion, this study successfully synthesized ZnO nanoparticles using a chemical reduction method and characterized their crystalline structure and morphology. Through Taguchi method analysis, it identified concentration as the most influential factor in enhancing Ghaf seed germination, followed by temperature, time, and agitation. The regression model developed based on these factors accurately predicted the percentage of responded seeds. These findings underscore the importance of nanoparticle concentration in optimizing seed germination and highlight the efficacy of the Taguchi method in systematically exploring and optimizing incubation parameters. Moreover, the experimental validation confirms the reliability and accuracy of the developed regression model, offering valuable insights into the interplay of various factors in seed germination enhancement. Ultimately, this research contributes to advancing seed priming protocols using ZnO nanoparticles, with implications for sustainable agricultural practices in arid environments. However, it is essential to acknowledge the study's limitations. Firstly, while the Taguchi method efficiently explores interactions among factors, further studies could incorporate additional variables that may influence seed germination, such as light conditions or soil pH. Secondly, the focus on ZnO nanoparticles warrants consideration of potential ecological impacts and long-term effects on soil health. Future research could delve into these aspects to ensure sustainable agricultural practices. Additionally, expanding the study to different geographical locations and varying environmental conditions would enhance the generalizability of our findings. By addressing these aspects, future studies can build upon our findings and contribute to further advancements in seed priming methodologies using nanotechnological approaches.

## Materials and methods

### Method of ZnO nanoparticle synthesis

ZnO nanoparticle was synthesized via a chemical reduction method^39^. Zinc nitrate tetrahydrate (Zn(NO_3_)_2_·4H_2_O) served as the precursor, with trisodium citrate dihydrate (Na_3_C_6_H_5_O_7_·2H_2_O) (TSC) as the capping agent and NaOH as the reducing agent. A 0.2 M Zinc nitrate solution was prepared in 100 mL ultrapure water, and a corresponding 0.2 M TSC solution was created. The two solutions were mixed, heated to 80 °C, and stirred. In order to induce the formation of a white precipitate, 0.2 M NaOH was gradually add under stirring (50 rpm). After 3 h of stirring, the solution cooled to room temperature. The resulting precipitate underwent washing with ultrapure water three times, centrifugation at 5000 rpm for 5 min, and an additional rinsing with absolute ethanol.

Drying occurred at 70 °C for 8 h, followed by grinding for uniformity. Crystalline ZnO nanoparticle was obtained through calcination at 450 °C for 2 h. This nanoparticle was then subjected to, X-ray diffraction analysis was conducted using a Bruker diffractometer (AXS Kappa APEX II CCD X-ray), operating at 40 mA and 40 kV, with Cu Kα radiation (1.54 Å) as the source. The obtained XRD patterns were compared using JPCDS standard card No: 043–0002. Transmission electron microscopy (TEM JOEL 2100) and scanning electron microscopy (JEOL JSM-7600F FEG-SEM) analysis. The average ZnO grain size was calculated using the full width at half maximum (FWHM) of diffraction curves by the Debye–Scherrer formula (3).

3 $$D=\frac{k\lambda }{\beta cos\theta },$$

where D is the particle size; λ—wavelength of X-ray radiation (0.15406 nm); K -Scherrer constant (0.9); β- full width at half maximum (FWHM) of the XRD peak; θ – Bragg’s angle^40^.

### Plant material

The 10 g of Ghaf seeds were surface sterilized by using 100 mL of 0.1% sodium hypochlorite for 2 min, followed by a thorough triple rinse with distilled water and were gently air-dried on blotting paper at a consistent room temperature of 22 ± 1 °C. These prepared seeds were subjected to runs designed by Taguchi OA L16 according to Morfidan et al.^41^.

### The experimental design using Taguchi approach

Taguchi approach was employed to select combinations of multiple factors for simultaneous experimentation on Ghaf responded seeds. The experimental design, utilizing Minitab 19.0 software, takes a systematic and multifactorial approach to comprehend the influence of ZnO nanoparticles on seed responsiveness.

#### Identification of factors and their respective levels

A Taguchi orthogonal array experimental design^41^ was employed, incorporating four key factors (concentration, temperature, time, and agitation), each at four distinct levels as given in Table 7. For study the concentration (125, 250, 500, 1000 ng/ml). Temperature (25, 30, 35 40 °C). Time durations selected were (2, 4, 8, 16 Hrs) and agitation were (0, 50,100, and 150 RPM). These factors were chosen to encompass a range of conditions that could significantly influence the responsiveness of Ghaf seeds to the ZnO nanoparticle treatment. Four level four factor leads to the L16 runs in triplicates given in Table 2.

**Table 7:** Incubation parameters and their levels selected for the optimization of Ghaf seeds germination using Taguchi method.

Incubation parameters	Abbreviation	Levels			
		1	2	3	4
Concentration (ng/mL)	C	125	250	500	1000
Temperature (ºC)	T	25	30	35	40
Time (Hr)	t	2	4	8	16
Agitation (RPM)	A	0	50	100	150

The optimization criterion for the experiments is the larger-the-better S/N ratio, aligning with the objective of maximizing the germination response^42^. The formula utilized for this assessment is defined (4)^43^.

4 $$ {\text{S}}/{\text{N}} {\text{ratio}} = - {1}0 \times {\text{log1}}0\,\left( {{\text{n}}\sum \left( {{1}/{\text{Y2}}} \right)} \right), $$

Y represents the measured response or output for each specific experimental run, while n denotes the total number of observations.

#### Analysis of variance

Analysis of variance (ANOVA) was employed using the Taguchi orthogonal array (OA) methodology to identify the most and least influential parameters affecting the enhancement of Ghaf seed germination rates^44^.

#### Regression model development

Regression analysis, a pivotal component in discerning the relationships between the response variable (germination rate) and selected incubation parameters, was carried out to develop predictive mathematical models^45^. In this investigation, linear regression analysis was utilized, implemented through statistical software (e.g. Minitab 19.0)^46^. The chosen input parameters for the regression models included concentrations of ZnO nanoparticles, treatment duration, temperature, and agitation levels during the seed priming process.

#### Contour plot and confirmatory analysis

Contour plots were created using the Taguchi approach to explore the intricate relationships between each pair of conditions, providing a visual representation of their interrelation^47^. To validate the findings, a confirmation test was executed by conducting an experiment with the predicted optimum levels for each condition, as determined by the program. The experiment was replicated three times, and the results were analyzed to calculate the error rate of the conducted experiment.

## Data Availability

The datasets generated during the current study are available from the corresponding author on reasonable request.

## References

[1] Alshehi A (2015). Filling the Empty Quarter: Declaring a Green Jihad on the Desert.

[2] Hassan FE (2023). Effective priming techniques to enhance Ghaf (*Prosopis*
*cineraria* L. Druce) seed germination for mass planting. Horticulturae.

[3] Chachalis D, Reddy KN (2000). Factors affecting *Campsis radicans* seed germination and seedling emergence. Weed Sci..

[4] Baibout M (2022). Ecosystem services provided by *Prosopis*
*cineraria* (L.) Druce in the drylands of Southern and Western Asia. Bot. Lett..

[5] Hassan FA (2023). Enhancing germination of Ghaf seeds (*Prosopis*
*cineraria* L.) using sulfuric acid scarification and cytokinin. Acta Hortic..

[6] Brown PH, Zhao F-J, Dobermann A (2022). What is a plant nutrient? Changing definitions to advance science and innovation in plant nutrition. Plant Soil.

[7] Waqas M, Hasanuzzaman M, Fotopoulos V (2019). Advances in the concept and methods of seed priming. Priming and Pretreatment of Seeds and Seedlings.

[8] Dawood MG, Rakshit A, Singh HB (2018). Stimulating plant tolerance against abiotic stress through seed priming. Advances in Seed Priming.

[9] Tian Y (2014). Responses of seed germination, seedling growth, and seed yield traits to seed pretreatment in maize ( *Zea*
*mays* L.). Sci. World J..

[10] Francis DV, Sood N, Gokhale T (2022). Biogenic CuO and ZnO nanoparticles as nanofertilizers for sustainable growth of *Amaranthus hybridus*. Plants.

[11] Farhangi H (2023). Optimizing growth conditions in vertical farming: enhancing lettuce and basil cultivation through the application of the Taguchi method. Sci. Rep..

[12] Das PP, Chakraborty S (2023). Optimization of friction stir welding processes using hybrid-taguchi methods: A comparative analysis. Int. J. Interact Des. Manuf..

[13] Barman K, Chakraborty P, Samanta PK (2021). Green synthesis of zinc oxide nanostructure using *Azadirachta Indica* leaf extract and its structural and microstructural study. Phys. Scr..

[14] Basak M (2022). The use of X-ray diffraction peak profile analysis to determine the structural parameters of cobalt ferrite nanoparticles using Debye-Scherrer, Williamson-Hall, Halder-Wagner and size-strain plot: Different precipitating agent approach. J. Alloys Compd..

[15] Sarıkaya M, Yılmaz V, Dilipak H (2016). Modeling and multi-response optimization of milling characteristics based on Taguchi and gray relational analysis. Proc. Inst. Mech. Eng. B J. Eng. Manuf..

[16] Venkatanarayana B, Ratnam Ch (2019). Selection of optimal performance parameters of DI diesel engine using Taguchi approach. Biofuels.

[17] Luangpaiboon P, Boonhao S, Montemanni R (2019). Steepest ant sense algorithm for parameter optimisation of multi-response processes based on taguchi design. J. Intell. Manuf..

[18] Pattnaik S, Sutar MK (2021). Advanced Taguchi-neural network prediction model for wire electrical discharge machining process. Process. Integr. Optim. Sustain..

[19] Maazinejad B (2020). Taguchi L9 (34) orthogonal array study based on methylene blue removal by single-walled carbon nanotubes-amine: Adsorption optimization using the experimental design method, kinetics, equilibrium and thermodynamics. J. Mol. Liquids.

[20] Mustafai FA (2018). Microwave-assisted synthesis of imprinted polymer for selective removal of arsenic from drinking water by applying Taguchi statistical method. Eur. Polym. J..

[21] Goienetxea Uriarte A (2017). How can decision makers be supported in the improvement of an emergency department? A simulation, optimization and data mining approach. Oper. Res. Health Care.

[22] Shi Z, Sun X, Cai Y, Yang Z (2020). Robust design optimization of a five-phase PM hub motor for fault-tolerant operation based on Taguchi method. IEEE Trans. Energy Convers..

[23] Santhosh AJ, Lakshmanan AR (2016). Investigation of ductile iron casting process parameters using Taguchi approach and response surface methodology. China Foundry.

[24] Anbazhagan G (2022). An effective energy management strategy in hybrid electric vehicles using Taguchi based approach for improved performance. Energy Sources A Recov. Util. Environ. Effects.

[25] Abou-Taleb NH (2021). Spider diagram and Analytical GREEnness metric approach for assessing the greenness of quantitative 1H-NMR determination of lamotrigine: Taguchi method based optimization. Chemom. Intell. Lab. Syst..

[26] El-Moslamy SH (2017). Applying Taguchi design and large-scale strategy for mycosynthesis of nano-silver from endophytic *Trichoderma*
*harzianum* SYA.F4 and its application against phytopathogens. Sci. Rep..

[27] Baz H (2020). Water-soluble carbon nanoparticles improve seed germination and post-germination growth of lettuce under salinity stress. Agronomy.

[28] Vera-Reyes I, López-Valdez F, Fernández-Luqueño F (2018). Effects of nanoparticles on germination, growth, and plant crop development. Agricultural Nanobiotechnology.

[29] Huang Z (2020). Response of rice genotypes with differential nitrate reductase-dependent NO synthesis to melatonin under ZnO nanoparticles’ (NPs) stress. Chemosphere.

[30] Kandhol N (2022). Nano-priming: Impression on the beginner of plant life. Plant Stress.

[31] Ragab G, Saad-Allah K (2021). Seed priming with greenly synthesized sulfur nanoparticles enhances antioxidative defense machinery and restricts oxidative injury under manganese stress in *Helianthus*
*annuus* (L.) seedlings. J. Plant Growth Regul..

[32] Acharya P (2020). Nanoparticle-mediated seed priming improves germination, growth, yield, and quality of watermelons (*Citrullus lanatus*) at multi-locations in Texas. Sci. Rep..

[33] Francis DV, Asif A, Ahmed ZFR, Khan M, Chen JT (2024). Nanoparticle-enhanced plant defense mechanisms harnessed by nanotechnology for sustainable crop protection. Nanoparticles in Plant Biotic Stress Management.

[34] Sarkhosh S (2014). Effect of zinc oxide nanoparticles (ZnO-NPs) on seed germination characteristics in two brassicaceae family species: *Camelina sativa* and *Brassica napus* L. J. Nanomater..

[35] Francis DV, Aiswarya T, Gokhale T (2022). Optimization of the incubation parameters for biogenic synthesis of WO3 nanoparticles using Taguchi method. Heliyon.

[36] Das P (2014). Optimization of degree of sphericity of primary phase during cooling slope casting of A356 Al alloy: Taguchi method and regression analysis. Measurement.

[37] Venkatesan G (2015). Regression analysis of a curved vane demister with Taguchi based optimization. Desalination.

[38] Yi H, Srinivasan RS, Braham WW (2015). An integrated energy–emergy approach to building form optimization: Use of EnergyPlus, emergy analysis and Taguchi-regression method. Build. Environ..

[39] Zhang L (2014). Shape-controlled synthesis of ZnO microstructures: The effects of inorganic shape directing and pH altering agents. J. Nanosci. Nanotechnol..

[40] Goswami M, Adhikary NC, Bhattacharjee S (2018). Effect of annealing temperatures on the structural and optical properties of zinc oxide nanoparticles prepared by chemical precipitation method. Optik.

[41] Mofidian R (2019). Optimization on thermal treatment synthesis of lactoferrin nanoparticles via Taguchi design method. SN Appl. Sci..

[42] Yang Z, Niu B, Pan Y, Chen Y (2023). Multi-objective optimization of supply air jet enhancing airflow uniformity in data center using Taguchi/CRITIC/TOPSIS triple method. Build. Environ..

[43] Haq AN, Marimuthu P, Jeyapaul R (2008). Multi response optimization of machining parameters of drilling Al/SiC metal matrix composite using grey relational analysis in the Taguchi method. Int J Adv Manuf Technol.

[44] Chen W-H (2022). A comprehensive review of thermoelectric generation optimization by statistical approach: Taguchi method, analysis of variance (ANOVA), and response surface methodology (RSM). Renew. Sustain. Energy Rev..

[45] Morales B, Kaskar O, Grace LR (2018). Design and processing of an elastomeric nanocomposite for biomedical pressure sensing applications. Mater. Today Commun..

[46] Benterki S (2023). Evaluation and optimization of erosion parameters’ effects on polymeric glasses using Taguchi method. J. Mater. Eng. Perform..

[47] Winarni S, Sunengsih N, Ginanjar I (2021). Multi responses Taguchi optimization using overlaid contour plot and desirability function. J. Phys. Conf. Ser..

